# An Alternative Treatment Option for Blauth III B Thumb Hypoplasia—Thumb Stabilization with Iliac Crest Bone Graft and Intermetacarpal Arthrodesis

**DOI:** 10.3390/jcm12185977

**Published:** 2023-09-15

**Authors:** Simon Oeckenpöhler, Martin Franz Langer, Anna Wichmann, Johannes Glasbrenner, Oliver Riesenbeck

**Affiliations:** Department of Trauma, Hand and Reconstructive Surgery, University Hospital Münster, Waldeyer Str. 1, 48149 Münster, Germany; simon.oeckenpoehler@ukmuenster.de (S.O.); anna.wichmann@ukmuenster.de (A.W.); johannes.glasbrenner@ukmuenster.de (J.G.); oliver.riesenbeck@ukmuenster.de (O.R.)

**Keywords:** thumb hypoplasia, preaxial longitudinal deficiency, Blauth, congenital

## Abstract

Thumb hypoplasia modified Blauth III B is usually treated by pollicization or, less commonly, by toe transfer. Both procedures always result in the resection of a body part, but with good cosmesis and acceptable function. We describe an intermetacarpal I/II arthrodesis with autologous bone graft augmentation to lengthen and stabilize the loose thumb. Clinical data were collected from nine patients, median age at surgery 3 years 8 months, with more than 7 years of follow-up. The results showed a grip strength on the Jamar dynamometer of approximately 61% of the unoperated hand. The Quick-DASH score was 11. The reconstructed thumb was 0.8 cm thinner and 1.9 cm shorter. Overall satisfaction on the VAS, with an average of 1.5 out of 10, is excellent with a partially usable thumb on a hand with five rays. The described procedure is a reliable treatment option with satisfactory results. In addition, none of the patients lost pincer grip between the second and third digit, but their thumb gained new function. Especially in environments where physical integrity has a high value, thumb construction instead of replacement could be considered.

## 1. Introduction

The thumb is of outstanding importance for the use of the hand as it allows precise and powerful grasping. Its capacity to oppose, which is unique to humans, is just as important [1]. Given the importance of a functional thumb, the wide spectrum of thumb hypoplasia is a clinical pattern that has led to many therapeutic approaches.

Thumb hypoplasia can range from a mild form where the thumb is reduced in size to extreme forms where the thumb is completely missing [2]. Hypoplasia can be spontaneous, associated with other diseases and syndromes, such as VACTERL association [3], or hereditary [4].

The common classification for thumb hypoplasia is the Blauth classification [5], which divides thumb hypoplasia into five grades. Grade I shows only mild hypoplasia in which all musculoskeletal and neurovascular components are present. Grade II is instability of the CMC I joint and thenar hypoplasia. Grade III is characterized by partial aplasia of the first metacarpal. Grade IV is complete aplasia of the first metacarpal, with only the proximal and distal phalanx remaining. Grade V includes any complete aplasia of the entire thumb. Several updates and modifications to the Blauth classification have led to the division of grade III into a IIIa and IIIb type [6,7]. IIIa patients have only abnormalities of the extrinsic muscles and tendons with a stable CMC-I joint, whereas IIIb patients also have a hypoplastic first metacarpal. Our study focused on patients with IIIb Blauth hypoplasia, whose thumbs could not perform any relevant function due to high instability.

First grade hypoplasia does not usually require surgery. For second and IIIa grade hypoplasia, reconstructive therapy is the most common choice [8,9,10]. For grade IIIb, IV and V, amputation of the hypoplastic thumb and subsequent pollicization of the index finger or toe transfer are probably the most commonly performed surgical therapies [11,12].

Modified Blauth IIIB thumb hypoplasia is characterized by the absence of the proximal part of the first metacarpal. This means that the first carpometacarpal joint is also absent [12]. The standard treatment for this type of hypoplasia, or more severe forms of thumb hypoplasia, is pollicizing the index finger [13]. This method uses the index finger instead of the thumb and was first developed by Littler in the 1950s Buck-Gramcko after the Thalidomide scandal in the 1960s and 1970s [14]. Another treatment option for mod. or severe Blauth IIIb is toe transfer. This option is usually chosen by patients and their families who want to keep a five-fingered hand for cultural, religious or aesthetic reasons [15]. In some cases of mod. Blauth IIIB thumb hypoplasia, the index finger is also affected with stiff joints and less developed extensor and flexor muscles. A pollicization of those index fingers results in inferior clinical results of pollicization.

Preaxial longitudinal deficiencies resulting in hypoplasia of the thumb in mod. or lesser Blauth IIIB are most commonly treated by pollicizing the index finger or, less commonly, by toe transfer. Both operations always result in the resection or “loss” of a body part, but with good cosmesis and acceptable function. In our opinion, the resection of a thumb, which the patient has some control of, is a waste of potential. In addition, in some cultures it is frowned upon to have a physical defect such as a missing finger or toe. We present a different treatment option and its results. What is the outcome for patients with mod. Blauth IIIb hypoplasia of the thumb who have been treated with an intermetacarpal arthrodesis?

## 2. Materials and Methods

The study was conducted in accordance with the Declaration of Helsinki and approved by the ethical committee of the “Ärztekammer Westfalen-Lippe and the University of Münster” (protocol code EK 2020-413-f-S, 15 June 2020) for studies involving humans. We present a retrospective cohort analysis of 9 patients who underwent inter-metacarpal arthrodesis I/II in our clinic. The demographic data are shown in Table 1. A total of 11 patients underwent the procedure and we attempted to follow them retrospectively. The patients presented to our clinic relatively late. This is due to external pretreatments that were referred late and the fact that only a few clinics in our region perform stabilizing procedures at all. Of these, one had died at the time of follow-up, unrelated to the surgery, and another was unavailable. Thus, n = 9 patients were finally included. All patients had been operated on by the same surgeon.

In 1930, Foerster described extra-articular thumb arthrodesis as a treatment option for a patient whose thenar muscles were completely paralyzed after polio infection [15]. He fixed the thumb in an opposed position to provide a functional grip by grafting an autologous bone graft from the patient’s tibia between the MCP I and MCP II bones. This technique has since been used and modified to restore grip function in patients who have lost it due to paralysis or trauma to the hand. In our department, we have modified the Foerster arthrodesis to an intermetacarpal I/II arthrodesis with autologous bone graft augmentation to lengthen and stabilize the loose thumb, especially in children. Compared to other techniques described (see below), this technique is also well suited for older children (see Table 2).

### Surgical Technique

As usual, the operation is performed in the absence of blood with a tourniquet on and under magnifying glasses or partially under a microscope. The incision is made on the dorsoradial side of the hand to visualize the first and second metacarpals. First, the proximal part of the first metacarpal is dissected, and the bony surface is visualized. The fascia and fibrous tissue connecting the first metacarpal to the trapezium are then sharply removed. This allows spontaneous lengthening and distalization of the hypoplastic thumb by 10 to 20 mm (depending on patients’ age and severity). The soft tissues are a limitation to the lengthening of the thumb. In particular, the neurovascular bundles are a limiting factor to avoid circulatory disturbances or sensory deficits. The shaft of the second metacarpal is now dissected and the bone exposed. The size of the resulting space between the proximal second metacarpal and the distally pulled first metacarpal is then measured, which in our series varied between 20 mm and 40 mm.

A tricortical iliac crest span is then harvested in a classic manner. The incision is made parallel to the iliac crest. The fascia is incised and the musculature is pulled off distally. After thorough hemostasis, the outer wall of the iliac bone is exposed. To harvest the iliac bone, the bone resection is performed under the iliac crest below the growth plate. The shape of the graft should be approximately trapezoidal and a little larger than planned to have the opportunity of reshaping the bone before using it. Two patients did not need a pelvic bone graft because some locally harvested cancellous bone could be used to perform the arthrodesis.

**Figure 1:**
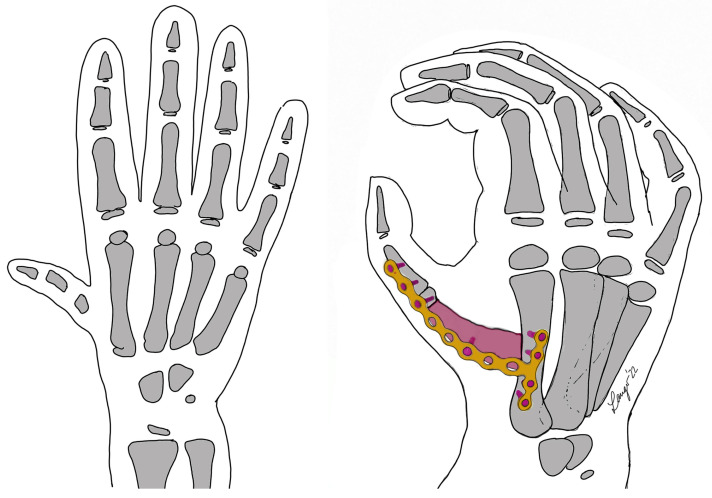
Schematic drawing of the procedure with iliac bone graft (pink) and plate osteosynthesis in a mod. Blauth III B hypoplastic thumb (**left side**).

**Figure 2:**
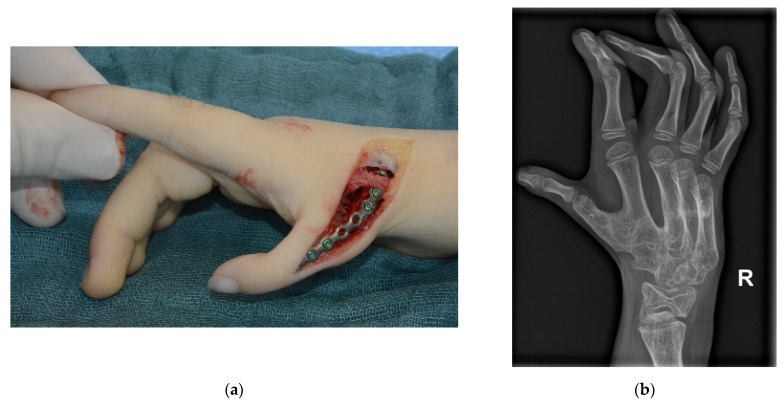
(**a**) Intraoperative picture of a bone graft interposition after plate fixation (**b**) X-ray of a long term follow up after bony consolidation and plate removal.

The aim of inserting the bone graft between the first and second metacarpal is to fix the thumb in opposition to the other fingers so that it can be used for grasping. The position of the thumb is adjusted to create an opposing grip to the index finger and the rest of the hand with a sufficient opening in the first commissure as shown in Figure 1. Fixation of the bone graft and the two metacarpal bones was achieved with plate osteosynthesis (1.2 to 2.0 mm plates, T-shaped and straight, manufactured by DePuy Synthes, Tuttlingen, Germany or Medartis, Basel, Switzerland, depending on the size of the patient) as shown in Figure 2a with a late follow up x-ray in Figure 2b. Depending on the size of first metacarpal, the inclusion of the proximal phalanx of the thumb in the arthrodesis was necessary to generate appropriate stability.

Finally, the gaps in the arthrodesis are filled with cancellous bone. After wound closure, all patients were immobilized with elastic and compression bandages. If necessary, patients received further immobilization, such as a cast that included the thumb or forearm, immobilizing the wrist and in between the metacarpals.

Patients were invited for follow-up after approval of the study by the Ethics Committee. Follow-up was performed by an investigator who was not involved in the surgery, postoperative care, or follow-up. Data for evaluation were collected once at a median of 7 years and 4 months after surgery.

Standardized clinical data including age at examination, sex, height, weight, age at primary surgery, number and type of secondary surgeries, comorbidities that could influence the results of the examinations, side of the operated hand, and hand dominance were collected.

**Figure 3:**
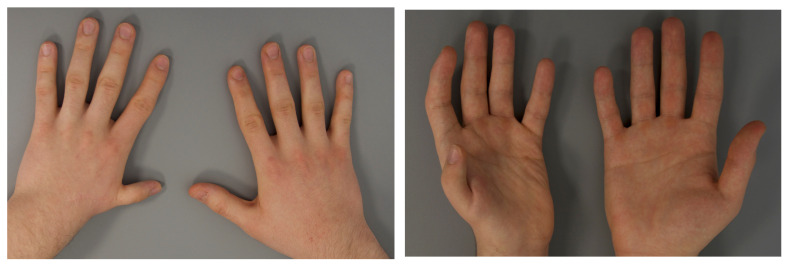
Dorso-palmar (**left**) and palmo-dorsal (**right**) pictures of both hands (left hand operated) of a patient at long term follow-up.

As part of the follow-up examination of both hands, several measurements were taken to objectively assess the position and size of the thumb. The following measurements were taken: extension of the thumb, length of the thumb from the base of the first metacarpal to the fingertip, length of the thumb from the commissure to the fingertip, difference in length to the index finger, maximum distance between the fingertip of the thumb and the fifth finger (handspan), and maximum distance between the fingertip of the thumb and the index finger. An example of a patient in follow-up is shown in Figure 3.

The Kapandji score was obtained to provide an objective idea of the resulting thumb reach. Functional data were collected using the Quick Disabilities of the Arm, Shoulder and Hand (Q-DASH) score [16,17]. Grip strength in both hands was measured using a Jamar hydraulic hand dynamometer (Performance Health Supply, Cedarburg, WI, USA). Patients overall subjective satisfaction was measured using a visual analog scale, with a score of 10 representing maximum satisfaction. Since all the patients in the study were over the age of 8, they were able to understand the question and respond appropriately.

To account for the fact that patients were of different ages both at the time of surgery and at the time of follow-up, data are always reported with reference to the contralateral side.

The Percival Score was developed by Percival et al. [18] to provide an objective assessment of the outcome of pollicization surgery. The test protocol includes several functional and cosmetic aspects. Since inter metacarpal arthrodesis was performed as an alternative treatment approach to pollicization in the present patient population, the Percival score was also determined here, to allow comparison with other studies, but with minor limitations or modifications, which will be explained. A major problem in congenital malformations of the thumb is the inability to hold objects between the thumb and the fingers, thus limiting pointer and pincer grasp. The Percival Score examines both the strength and accuracy of the pointed and pincer grips. Strength can be compared to either the (healthy) contralateral side or to a child of similar age and stature. For this work, the strength of the contralateral side was used as a reference. Accuracy is assessed by picking up a paper clip (pointed grip) or a key (pincer grip). In the case of the pointed grip, it is further subdivided into whether the task can be performed easily or with difficulty, while in the case of the pincer grip, a point is awarded if the patient can pick up the key without difficulty. Next, the ability to resist is assessed. In pollicization, the hypoplastic thumb is resected and the index finger is asked to perform the function of the thumb. This leaves only the three remaining fingers for opposition. The Percival score assigns one point for each possible opposition finger, for a maximum of three points. Since the patients in the present collective still have four fingers, the scoring scheme was adjusted accordingly. Instead of one point, 0.75 points are given for each finger, so the maximum number of points is still three, but all fingers are considered. Gripping ability is assessed by the ability to pick up a ping pong ball and a tennis ball, as well as by determining the strength of the grip, analogous to the measurement of the point and pincer grips. The mobility of the three joints of the pollicized index finger is also assessed. Due to the bony stiffening and the mostly absent or rudimentary musculature and ligaments, poorer results can be expected in patients with arthrodesis. The next step is to assess sensitivity. Static two-point discrimination is assessed. An Arex two-point discriminator is used for this purpose. Percival assigns three points for a “normal” two-point discrimination (≤5 mm), 2 points for a discrimination of 6–10 mm, and one point for a discrimination of >10 mm. Finally, the “aesthetic appearance” of the thumb or hand is assessed. Length, position, and subjective satisfaction are evaluated. For length, one point is awarded if the thumb ends within 0.5 cm of the proximal interphalangeal joint of the second finger. Since patients with inter metacarpal arthrodesis cannot place the thumb against the second finger, the length of the thumb is compared to the distance of the proximal interphalangeal joint of the second finger, measured from the commissure in each case.

The nine-hole peg test (NHPT) was first described by Kellor et al. [19] and later proposed by Mathiowetz et al. [20] as a standardized method for the assessment of manual dexterity. The advantages of the NHPT are its ease and speed of administration and its high interrater reliability. In the NHPT, the patient is instructed to sequentially insert nine dowels from a shallow container into designated holes in a plate and then sequentially return them to the container. The storage container is placed on the side of the hand to be tested and the plate with the holes is placed next to it. Time is measured from the moment the patient touches the first pin to the moment the last pin touches the storage container. The patient is always allowed a trial run. The time is then stopped, first for the unoperated hand, then for the operated hand. A test setup based on that described by Mathiowetz et al. was used for the study.

## 3. Results

The patients underwent a median of three operations, including primary surgery and implant removal. In eight of the nine patients, the course of therapy was free of complications. Only one patient experienced a plate failure, but this did not affect the consolidation of the arthrodesis. Seven patients later underwent a corrective osteotomy to improve the opposition. In one patient, the thenar was widened and stabilized by a dorsal transposition flap according to Dieffenbach. 

The median circumference of the operated thumb was 4.6 cm, 0.8 cm less than the contralateral thumb. The median length of the thumb, measured from the base of the MCP I to the tip, was 7.8 cm, 1.8 cm shorter than the unoperated thumb. The length from the commissure to the fingertip was median 3.5 cm and 1.5 cm shorter than the other thumb. The difference in length to the index finger was 4.9 cm, which was 1.0 cm more than the opposite hand. The median difference in hand span was 11.5 cm, 5 cm less than the contralateral hand. The maximum span between the thumb and the index finger was 8 cm, which was 3 cm less than on the contralateral hand.

The measurement of hand strength was possible for all patients. The median absolute strength on the operated side was 8 kg. Compared to the contralateral side, the patients with the operated hand were able to exert a median of 61.5% of the strength of the non-operated side; there was a large heterogeneity among the patients. To improve comparability, the normative values for age and gender are given in brackets [21,22] in Table 2. In this way, comparability was also achieved for patients with involvement of the opposite side. Compared to the normal value, the strength development was approximately 39%.

On the numerical rating scale for satisfaction, three patients gave the best possible rating (0/10), one patient rated her satisfaction at 1/10, five patients at 2/10 and one patient at 3/10. This results in an overall satisfaction rating of 1.5 out of 10, where 10 describes maximum dissatisfaction.

Pain was not reported by any of the patients.

Table 2 provides a summary of functional scores and patient demographics.

## 4. Discussion

In this study, nine patients who had undergone intermetacarpal arthrodesis were examined in a variety of ways. The amount of data is sufficient to allow comparison with other methods and studies.

However, there are limitations to the study: the case number of nine patients allows conclusions to be drawn about the complication rate and the reproducibility of the surgery. Nevertheless, the wide age distribution at the time of surgery, the various secondary diagnoses and the study design limit the validity of the study. Randomization is not possible in this sensitive disease, so a high level of evidence cannot be generated.

Kawabata et al. [23] studied 12 patients in whom the hypoplastic thumb was reconstructed with an avascular phalanx graft. The length of the thumb relative to the index finger was determined. The average length of the thumb was 57% of the length of the index finger. In our patient population, the thumb of patients with intermetacarpal arthrodesis was shorter than the index finger with a mean of approximately 43.5%, which is less than in Kawabata et al. Although the hypoplastic thumb can be lengthened by approximately 10 to 20 mm during arthrodesis therapy by cutting connective tissue strands (see Surgical Technique Section), it usually remains shortened compared to a normal thumb. On the other hand, after resection and transposition of a phalanx, there was no harvesting defect in our patients.

The difference between the hand span and the contralateral side in patients with intermetacarpal arthrodesis was highly variable and depended on the intraoperative opposition and position of the thumb, which cannot be changed postoperatively.

Patients in the cohort described above had an average Kapandji score of 3.1, meaning they were able to reach the ring finger. In comparison to Bachy et al. [24], who performed a follow-up of patients with pollicization and measured an average Kapandji score of 6, and Kollitz et al. [25], who reported an average Kapandji score of 6.4 in patients who had also undergone pollicization, the results described above are of course worse due to the fixed position of the thumb.

The patients described here were able to exert an average of 61.5% of the strength of the contralateral side with the operated hand. Grip strength in patients with treated thumb hypoplasia has been extensively documented in the literature. Most studies compared the strength of the operated hand with the normal contralateral hand and reported results between 60 and 70%. Netscher et al. [26], who studied patients with pollicization, reported that the strength of the operated hand was 60% of the contralateral hand, Chow et al. [27], who reconstructed the hypoplastic thumb with a hemilongitudinal metatarsal transfer, reported 61%, and Hu et al. [28], who stabilized the hypoplastic thumb by creating a pseudoarthrosis, reported 67%. Patients treated with pollicization in a cohort studied by Vekris et al. [29] had slightly more strength than the contralateral side with 76%, while patients treated with pollicization in a study by Staines et al. [30] developed significantly less strength in the operated hand with an average of 35%. From a small group of patients studied by Foucher et al. [31] who received thumb joint reconstruction with metatarsophalangeal joint transfer, only three of the five patients examined were able to operate the dynamometer at all, achieving 32, 42, and 44% of the strength of the opposite side. In summary, regardless of the treatment method chosen, the strength of the operated hand remains reduced compared to normal and compared to the contralateral side.

Since the Percival Score is a tool designed to evaluate patients who have undergone pollicization, it can only be partially applied to patients with inter-metacarpal arthrodesis due to the lack of mobility (see above for details). The average Percival score was only 10.8, which means that no patient achieved a result better than “satisfactory”. Hellevuo et al. [11] reported an average Percival score of 17 in their study of patients who had undergone pollicization. Vekris et al. [29] performed a 9-year follow-up of 21 patients who had undergone pollicization of the index finger and 75% had excellent satisfaction, 19% had good satisfaction and only 6% had poor satisfaction (all measured by the Percival score).

In the nine-hole peg test, patients in the arthrodesis group achieved a mean time of 43 s in the operated hand and a mean time of 21 s in the unoperated hand. Poole et al. [32] reported norm values in the same age group as our cohort (mean age 11.7 years) of 16.7 to 18.9 s in the dominant hand and 19 to 20.2 s in the non-dominant hand, which means that the operated hand requires significantly more time than the normal population, especially since our patient cohort achieved normal time levels in the non-operated hand.

Ince et al. [33] studied patients who underwent thumb amputation and secondary ring finger transfer for thumb reconstruction. The patients achieved a postoperative DASH score of 13.8 ± 7.5. Lahiji et al. [34] wrote a case series report of patients with a radial club hand who underwent pollicization and achieved a mean DASH score of 34.2 ± 9.7. The described arthrodesis cohort had a mean Quick-DASH score of 11.4, which is a much better result compared to other studies.

Accordingly, the function of the hand is inferior to that after intermetacarpal arthrodesis.

## 5. Conclusions

This study shows that intermetacarpal arthrodesis can provide a reliable and esthetically satisfactory reconstruction of the thumb. Overall, however, the functional tests showed the inferiority of this technique compared to other procedures such as pollicization.

However, it must be remembered that the patient retains the ulnar grasp between the index and middle finger, which is usually well developed at the time of pollicization. Furthermore, there is no harvesting defect in the hand and no surgery on the foot. There is also no risk of graft failure or loss of the transferred index finger. With this procedure, one still retains all the alternative options of secondary surgery with thumb replacement.

For patients or their parents who want a five-rayed hand for aesthetic, cultural or religious reasons, this may be also a good and reliable treatment option as well as the arthroplasty described by Kawabata [23] and Chow [27].

## Figures and Tables

**Table 1:** Patients demography and other data.

**Category**	**Patients (n = 9)**
Sex	4 female, 5 male
Median age at primary surgery	3 years and 8 months
Median age at follow-up examination	11 years and 11 months
Median follow-up time	7 years and 4 months
Patients with complications	1/9 (plate failure, but bony consolidation)

**Table 2:** Overview of demographic data, comorbidities and objective functional test results and scores (force (kg) = hand force measurement (grip in kg), Q-DASH Score = quick-DASH score, 9-Hole Peg = nine-hole peg test (s)).

	Age at Surgery	Age at Follow Up	Sex	Comorbidity	Kapandji Score	Force [kg] (Norm Value)	Percival Score	Q-DASH^®®^ Score	9-Hole Peg
1	1 yr. 9 mos.	9 yr. 1 mo.	f	Oligosyndactyly with one os metacarpale for the fourth and fifth finger and delta phalanx on the index finger, reduction defect with radiohumeral synostosis at the elbow	5	5 (15.1)	14.25	31.8	56
2	9 yr. 9 mos.	13 yr. 3 mos.	f		2	8 (24.5)	8.75	25	53
3	17 yr. 0 mos.	25 yr. 10 mos.	f	Thumb hypoplasia opposite side Blauth 1	3	21 (33.6)	8.75	2.27	17
4	6 yr. 4 mos.	8 yr. 1 mo.	m	Thumb hypoplasia opposite side Blauth 1	1	8 (15.9)	8.75	-	43
5	3 yr. 8 mos.	18 yr. 7 mos.	m		5	24 (48.3)	11.25	0	40
6	2 yr. 11 mos.	8 yr. 7 mos.	m	Radial aplasia with radial club hand	5	2 (14.6)	12.25	20.4	44
7	2 yr. 6 mos.	8 yr. 1 mos.	m	Bayne and Klug grade II radial hypoplasia	0	2 (14.6)	11.75	2.3	27
8	4 yr. 5 mos.	12 yr. 4 mos.	f	Thumb hypoplasia opposite side Blauth 1	4	10 (22.3)	12.5	2.3	26
9	3 yr. 2 mos.	11 yr. 11 mos.	m	Radial aplasia with radial club hand	3	4 (20.6)	8.75	38.6	89
Average					3.1	9.3	10.77	15.3	43.9
Median					3	8	11.25	11.4	43

## Data Availability

The data are not publicly available due to privacy of the included patients.
